# Thallium-induced DNA damage, genetic, and epigenetic alterations

**DOI:** 10.3389/fgene.2023.1168713

**Published:** 2023-04-20

**Authors:** Laura Sánchez-Chapul, Abel Santamaría, Michael Aschner, Tao Ke, Alexey A. Tinkov, Isaac Túnez, Laura Osorio-Rico, Sonia Galván-Arzate, Edgar Rangel-López

**Affiliations:** ^1^ Laboratorio de Enfermedades Neuromusculares, División de Neurociencias Clínicas, Instituto Nacional de Rehabilitación Luis Guillermo Ibarra Ibarra, Mexico City, Mexico; ^2^ Laboratorio de Aminoácidos Excitadores/Laboratorio de Neurofarmacología Molecular y Nanotecnología, Instituto Nacional de Neurología y Neurocirugía, Mexico City, Mexico; ^3^ Department of Molecular Pharmacology, Albert Einstein College of Medicine, Bronx, NY, United States; ^4^ Yaroslavl State University, Medical University (Sechenov University), Moscow, Russia; ^5^ Instituto de Investigaciones Biomédicas Maimonides de Córdoba, Departamento de Bioquímica Y Biología Molecular, Facultad de Medicina Y Enfermería, Red Española de Excelencia en Estimulación Cerebral (REDESTIM), Universidad de, Córdoba, Spain; ^6^ Departamento de Neuroquímica, Instituto Nacional de Neurología y Neurocirugía, Mexico City, Mexico

**Keywords:** thallium and DNA damage, thallium systemic toxicity, DNA alterations, cytogenetic damage, mutations, epigenetic changes, cancer

## Abstract

Thallium (Tl) is a toxic heavy metal responsible for noxious effects in living organisms. As a pollutant, Tl can be found in the environment at high concentrations, especially in industrial areas. Systemic toxicity induced by this toxic metal can affect cell metabolism, including redox alterations, mitochondrial dysfunction, and activation of apoptotic signaling pathways. Recent focus on Tl toxicity has been devoted to the characterization of its effects at the nuclear level, with emphasis on DNA, which, in turn, may be responsible for cytogenetic damage, mutations, and epigenetic changes. In this work, we review and discuss past and recent evidence on the toxic effects of Tl at the systemic level and its effects on DNA. We also address Tl’s role in cancer and its control.

## 1 Introduction

### 1.1 Thallium generalities

Thallium (Tl) is a ubiquitous natural trace metal discovered in 1861, considered one of the most toxic heavy metals to humans and animals. Since its discovery, Tl has been used in industrial applications as rodenticide, cement, insecticides, alloys, electronic devices, special glass, and optical lenses, in the industries of photography, semiconductors, and superconductivity (Taylor and McKillop, 1970). In addition, it has been used as a constituent of the growth media used for culture of some species of *Mycoplasma* (Hwang et al., 2010; Puschner et al., 2012). It is well documented that some anthropogenic activities such as the pyrite mining, smelting and related steel-making industry are also important source for Tl and other toxic metalloids pollution in the environment with the consequences in increasing the associated health risks (Wang et al., 2021). Unfortunately, Tl is still used as a rodenticide in many developing countries and in other novel technologies, raising concern about exposure risk to animals and humans. In the past, Tl has been employed for treating heavy persistent night sweats or ringworm (Galván-Arzate and Santamaría, 1998) as well as tuberculosis, sexually transmitted diseases such as syphilis and gonorrhea, for the production of depilatory products, as well as for homicidal purposes (Smith and Carson, 1977). Several decades ago, Tl isotopes have been used as radiotracers for cardiovascular and tumor imaging (National Minerals Information Center, 2022).

Thallium contributes to several health risk concerns (Liu et al., 2017) through accidental industrial occupational exposures such as coal burning and smelting (Smith and Carson, 1977; Brockhaus et al., 1981), environmental contamination, and therapeutic poisoning. Thallium is found in two oxidation states, thallous (Tl^+^) and thallic (Tl_3_^+^) salts. Because of its similarity to potassium (K^+^) ions, plants and animals readily absorb Tl^+^ through the skin and digestive and respiratory systems. When Tl is exposed to air and moisture, Tl oxidizes to form Tl(I) oxide (Tl_2_O) with chemical properties analogous to alkali metals, and when in its oxide form, at high temperatures it forms Tl(III) oxide (Tl_2_O_3_), behaving analogous to aluminum. Both of these species are highly toxic to living organisms (Rickwood et al., 2015), but Tl(I) is the predominant species. Thallium is found at low concentrations in the environment, and it is a trace compound for many minerals such as copper, gold, potassium, rubidium, and zinc. Some of the minerals with higher Tl concentrations (up to 16%–60% of Tl) are rookesite (found in Sweden and Brazil), lorandite (found in Greece and the United States), hutchinsonite (Switzerland), orabite (in Brazil and the United States), and berzelianite (in Germany) (Léonard and Gerber, 1997). There are few areas in the world with natural occurrence of high Tl concentrations, such as the southwestern Guizhou Province in China (Xiao et al., 2003), the Allchar area in Kavadarci, Macedonia (Percival and Radtke, 1994), and Lengenbach in Switzerland (Hofmann and Knill, 1996).

Thallium is released into the biosphere from both natural (volcanic activity and mineral ores) and anthropogenic (such as dust, vapors, or liquids from district heating, exhaust emissions, and coal combustion) sources, resulting in air pollution, thus allowing for its atmospheric spread and entry into terrestrial environment niches, binding to soil matrix (Savariar, 2014). Thallium is readily soluble in water and relatively more toxic than mercury, cadmium, lead, copper, or zinc (Cvjetko et al., 2010). The Tl-based salts are highly soluble, without color, odor, or taste, readily contaminating aquatic environments and accumulating in aquatic organisms. In addition, Tl compounds can leak into underground water streams or shallow soil and be taken up by roots leading to Tl accumulation in the plant biomass; therefore, once Tl has entered the food chain, it reaches living organisms with potentially toxic consequences (Karbowska, 2016; Liu et al., 2019). It has been demonstrated that some rivers and lakes downstream from coal mines and coal-fired power stations in Canada contain elevated Tl concentrations (Cheam et al., 2000; Cheam, 2001). Similarly, in North America and Europe, coal burning was determined as the main source of Tl, cadmium, and lead atmospheric pollution since the early 1900s, and the risk for increased levels of these elements is higher in the coal-driven economies (McConnell and Edwards, 2008).

Regarding some cases of intoxication, a recent study quantified the levels of Tl in urine from opium consumers developing symptoms similar to Tl poisoning. The study was carried out in the city of Kashan, in Iran, with 200 volunteers. Noteworthy, in the group of opium consumers, Tl concentrations reached an average of 38.93 μg/l, being significantly different from the control group (Eghtesadi et al., 2021). It was also reported in Malaysia that some baby diaper powders were contaminated with Tl^+^ and other heavy metals (Karwowski et al., 2017). In addition, unfortunately, Tl has also been used for war purposes as reported in Syria in 2015, where Tl-containing-candies were distributed among the population and were the cause of massive poisoning (Almassri and Sekkarie, 2018). Noteworthy, it has been determined that concentrations up to 0.43 μg/g of Tl^+^ have been found in tatoo inks obtained from some countries, hence constituting a health risk for the population who practice tattooing (Karbowska et al., 2020). Altogether, these and other cases demonstrate that more severe restrictions regarding the thallium and other metals pollution are needed (López Segura et al., 2013).

### 1.2 Systemic thallium toxicity

Since a biological function has yet to be demonstrated for Tl, it does not appear to be an essential element for humans or other living organisms (Cvjetko et al., 2010). One report in mice using field desorption mass spectrometry revealed that stomach was the organ showing the maximal levels of Tl accumulation, followed by kidney, heart, liver and brain during the first hours after exposure. Later, at 24 h after exposure, all organs (including the brain) were reported with higher accumulation (Achenbach et al., 1980). In rats, Tl was shown to accumulate in a higher concentration in kidney, followed by testis, spleen, lung, heart, liver and brain (Ríos et al., 1989). In human beings, Tl can be accumulated in several organs, and the resulting symptomatology is non-specific due to the multi-organ involvement, though the highest concentration has been reported in kidney, followed, in decreasing order, by bones, stomach, intestin e, spleen, liver, muscle, lung, and brain (Léonard and Gerber, 1997); the severity of intoxicat ion and t issue damage depends on the amount of Tl incorporated, age, the immune response, and each person’s tolerance to this poisoning element (Misra et al., 2003).

Regarding Tl toxic mechanisms, it has been suggested that thallium toxicity is related to the interference with relevant potassium-dependent processes, such as the (Na^+^/K^+^)^-^ ATPase pump, since Tl^+^ and K^+^ are both univalent ions with similar ionic radii (173 pm and 165 pm respectively). In this regard, Tl^+^ ions are capable to mimics K^+^, therefore modifying the potassium cellular uptake and intracellular accumulation in mammals (Britten and Blank, 1968; Mulkey and Oehme, 1993; Galván-Arzate and Santamaría, 1998). Chen et al. (2000) demonstrated that Tl^+^ and K^+^ ions behave as analogous ions using murine-cultured rat cells. Authors reported that the previous treatment with extracellular K^+^ significantly evoked a reduction of thallium-201 ion influx in these cells, whereas the treatment with ouabain, (an inhibitor of sodium-potassium ATPase pump), diminished the uptake of thallium-201 ion in these cells via active transport.

Thallium intoxication begins after this metal is introduced into the body by inhalation, ingestion of contaminated food or through the skin. Water-soluble Tl^+^ salts are able to enter in organisms and are widely distributed in organs and tissues, including the brain, heart, kidney, skeletal muscle and testis (Mulkey and Oehme, 1993; Galván-Arzate and Santamaría, 1998). Once Tl enters the body through the potassium uptake mechanisms, it is distributed throughout the organism, and accumulates intracellularly, where it may affect the functionality of various tissues and systems (Heim et al., 2002; Amiri et al., 2007; Fatemi et al., 2007; Osorio-Rico et al., 2017; Campanella et al., 2019).

Though the precise mechanisms of Tl toxicity remain unknown, its damaging effects have been related to: a) binding to blood proteins (Xiao et al., 2004; Peter and Viraraghavan, 2005); b) increase in the levels of reactive oxygen species (ROS) -which represents an important riskfactor for tissue injury and dysfunction (Radic et al., 2009)-; c) interference with the function of membrane channels and transporters associated with potassium (K^+^) (Kilic and Kutlu, 2010), induction of mitochondrial dysfunction -which interferes with vital potassium-dependent processes, disturbing the levels and expression of the Na^+^-K^+^-ATP enzymes inside cells (Zhao et al., 2008)-; d) triggering metabolic disturbances such as the inhibition of enzymes, coenzymes and structural proteins - which in turn could explain the structural changes observed in the affected cells that may correlate with physical, psychiatric, and neurological abnormalities present in these patients (Galván-Arzate and Santamarıa, 1998; Bragadin et al., 2003; Korotkov and Lapin, 2003)-; and e) inhibition of cellular respiration and disruption of calcium homeostasis when Tl forms complexes with proteins containing sulfhydryl groups (Kilic and Kutlu, 2010).

The estimated lethal dose for oral administration is between 10 and 15 mg/kg (Galván-Arzate and Santamaría, 1998; Conerly and Blain, 2008; Lennartson, 2015), sufficient to cause fever and gastrointestinal problems which appear within the few hours of acute poisoning, as well as seizures, memory impairment, mental disturbances, coma, and delirium (Saddique and Peterson, 1983). Reed et al. (1963) performed a 7-year study demonstrating that more than seventy children who ingested thallium-containing pesticides suffered neurological, gastrointestinal dysfunctions, and cardiovascular abnormalities. They estimated that renal excretion of thallium sulfate was slow, and this salt could be detected as late as 2 months after its ingestion. The biological half-time of Tl permanence in humans has been estimated between 10 and 30 days (World Health Organization WHO, 1996), with a mortality rate of 6%–15% (Ratti et al., 2018); therefore, it is important to determine an early diagnosis as to establish the most efficacious treatment.

Thallium (80%–100%) is readily absorbed through the skin and mucous membranes. The most common routes of exposure are via the gastrointestinal or pulmonary mucous by consuming contaminated food and drinking water or being exposed to air-borne contamination (Liu et al., 2020). During acute exposure, gastrointestinal symptoms such as severe abdominal pain, nausea, vomiting, and bloody diarrhea, appear within the first few hours of poisoning; whereas in chronic exposure the main symptoms are chest tightness, confusion, coma, and dyspnea, resulting in poor prognosis (Xu et al., 2010). These patients can also present non-specific skin abnormalities such as scaling of the palms and soles, acneiform lesions of the face, numbness, burning pain, hypertension, tachycardia, and hyperalgesia (Zhao et al., 2008). During chronic exposure, the main neurological symptoms include sensory and motor dysfunctions (Saddique and Peterson, 1983).

Clinical manifestations depend on the level of Tl intoxication, severity (acute, chronic, or sub-chronic), time of exposure, age and the general health status. Unfortunately, the lack of non-specific symptoms at the beginning of intoxication delays adequate diagnosis and decisions on appropriate therapeutic measures (Lech and Sadlik, 2007); nonetheless, the main symptom of Tl poisoning is alopecia, which appears on day 10–14, sometimes accompanied by loss of eyebrows and eyelashes, forcing the poisoned persons to contact a physician (Saddique and Peterson, 1983; Misra et al., 2003). The high affinity of Tl for sulfhydryl groups in proteins and other biomolecules may explain alopecia as Tl prevents keratinization of hair proteins by binding with cysteine residues, thus inducing atrophic and necrotic changes, which in turn, generate parakeratosis, necrotic sebaceous materials, dilated hair follicles filled with keratin, vascular degeneration of basal layer, and mild epidermal atrophy (Saha et al., 2004; Kuo et al., 2005; Jha et al., 2006; Lu et al., 2007). Approximately 1 month after Tl poisoning, the characteristic Mee’s lines appear in the nail plate of exposed people; these lesions manifest as transverse white lines presented as a result of complete erosion of the proximal parts of nails (Zhao et al., 2008).

Thallium can readily cross the blood-brain barrier leading to neurodegeneration, demyelination, and accumulation of end products of lipid oxidation in the brain of animal models (Galván-Arzate et al., 2000). Periodically, Tl poisoning is misdiagnosed as Guillain-Barré syndrome as some symptoms of neuropathy with muscle weakness, paresthesia, and hyporeflexia are detected (Jha et al., 2006). Also, some peripheral neuropathies with severe pain have been reported, accompanied by paresthesia of hands and lower limbs, distal motor weakness, and sensory impairment (Kuo et al., 2005). Thallium can also readily cross the placental barrier, and excreted in breast milk (Hoffman, 2000). During pregnancy, mothers exposed to Tl intoxication develop classic signs of thallium poisoning; however, their newborns appear normal and apparently, the only consistent reported effect is prematurity and low birth weight, despite the potential for severe maternal toxicity (Hoffman, 2000). Unfortunately, there are still insufficient data to evaluate prognostic markers of fetal outcome and the benefits or risks of treatment in Tl-poisoned pregnant patients.

Thallium is released slowly from the organism through sweat, saliva, tears, urine, and feces, and it is accumulated in the hair and nails. Elevated Tl levels in urine, saliva, and blood are better indicators of poisoning than its presence in hair, as Tl in hair could be explained by exogenous deposition (Tl-containing dust) (Frattini, 2009).

The misdiagnosis or delayed diagnosis leads patients to develop unfavorable outcomes (Hoffman, 2003; Jha et al., 2006; Lin et al., 2019) or severe or moderate sequelae of poisoning in sensory nerve fibers of the distal lower extremity and evokes problems in intelligence, depression, and memory. When hospital admission is delayed, it may lead to fatalities (Sun et al., 2012; Li et al., 2014).

### 1.3 Animal models

Tl-induced mechanisms of toxicity have been poorly studied and understood in living organisms due to their extreme toxicity. Therefore, only limited mutagenicity, genotoxicity, and epidemiological data to classify Tl carcinogenic potential have been reported (Aresini et al., 1993). Most of these reports have been focused on studying the efficacy of treatments for experimental thallium poisoning. Given that liver is the preferred site of Tl storage, it has been reported that Tl administration in a rat experimental liver injury model induced mitochondrial membrane potential collapse in isolated rat hepatocytes (Eskandari et al., 2015), which was evidenced by the impairment of mitochondrial fatty acid metabolism (Li et al., 2022). Using an *in vitro* model for studying the mitochondrial effects of Tl exposure, Eskandari and others showed a marked increase in oxidative stress characterized by elevated mitochondrial ROS generation, ATP depletion, oxidation of reduced glutathione (GSH), and mitochondrial membrane potential collapse, accompanied by mitochondrial outer membrane rupture and swelling and disruption of the mitochondrial respiratory chain. Combined, these effects triggered hepatocellular death secondary to the opening of the mitochondrial permeability transition pore (Eskandari et al., 2015).

## 2 Cellular effects of thallium exposure

Because of its similarity to potassium ions, Tl cannot only be absorbed through the skin, digestive, and respiratory systems, but also cross the placental, hematoencephalic, and gonadal barriers. Once the thallium ion (Tl^+^) reaches intracellular compartments, it can accumulate and interfere with the metabolism of potassium and other metal cations, mimicking or inhibiting their action. The toxicity of Tl compounds is predominantly related to their affinity for amino-, imino-, and sulfhydryl groups at catalytic enzymatic sites, similar to mercury, which possesses an affinity for selenol and sulfhydryl groups of bioligands (Rodríguez-Mercado and Altamirano-Lozano, 2013; Ajsuvakova et al., 2020).

Hantson et al. (1997) reported some preliminary observations showing that Tl can induce cytogenetic damage *in vivo* when analyzing samples of peripheral blood lymphocytes from a patient who self-ingested a dose of 200 mg of thallium sulfate. These authors reported drastic increases in binucleated cells presenting micronuclei, suggesting that Tl2SO4 interferes more with chromosome distribution than with the structural chromosome integrity or the sister chromatid exchange process.

Rodríguez-Mercado et al. (2013) determined *in vitro* that increased concentrations of thallium(I) acetate induced a concentration-dependent reduction in the mitotic and replicative indices in human peripheral blood cells when assayed for structural chromosomal aberrations and sister chromatid exchanges. Additionally, increased structural chromosomal aberrations were observed in the Tl treated group compared with the control group.

Hanzel et al. (2012) demonstrated that Tl triggered apoptosis. Here, the authors treated PC12 cells for 3 h with 100 μM Tl(III) and observed marked endosomal acidification content characterized by altered profile of acridine orange uptake. This was associated with increased levels of Cathepsins B and D activities and decreased the co-localization of these enzymes with the lysosomal marker Lamp-1. The authors hypothesized that Tl may be taken up via iron uptake mechanisms, in turn, leading to lysosomal functions. Notably, this group observed that Cathepsin D activated the pro-apoptotic protein BID, which is involved in the intrinsic pathway of apoptosis. When PC12 cells were preincubated with pepstatin A, a cathepsin D inhibitor, further treatment with Tl(III) did not activate caspase 3 and therefore, apoptosis progression was blocked. These results support the concept that Tl(III) is incorporated by the acidic cell compartments and the role played in the early steps of Tl-mediated apoptosis in PC12 cells.

Rodríguez-Mercado and Altamirano-Lozano (2013) also reported that Tl induced cytotoxic and mutagenic effects, as well as DNA breaks, reduced cell viability, interferes with the cell cycle progression, induced apoptosis, inhibited DNA synthesis, and blocked cell proliferation in a variety of mammalian cells such as embryo fibroblasts, osteoblasts, hepatocytes, glioma cells, ovarian cells, and lymphocytes from mice, rats, Chinese hamsters, and human cells. In an elegant review, these authors discussed several cell lines and animal models such as C57B1/6 strain, CBA strain, 3T3 cells, MC3T3-E1 cells, L929 cells, rat and human primary hepatocytes, C6 glioma cells, Chinese hamster’s ovary cells, lymphocyte cultures, HepG2 cells, Jurkat cells, and Chinese hamster’s bone marrow, all of which have been used to elucidate the mechanism of Tl-induced cellular damage.

Recently, it was shown that human peripheral lymphocytes treated with increased concentrations (0.5–100 μg/mL) of thallium (I) acetate, thallium (I) sulfate, and thallium (III) chloride had decreased viability secondary to accelerated apoptosis and necrosis in a concentration and time-dependent manner. When the cells were treated with these 3 Tl compounds in the G0 phase, the mitotic index was diminished in a concentration-dependent manner, suggesting that cell membrane is the main target of Tl. In addition, it was observed that these Tl salts decreased the cellular proliferative rate, characterized by dysfunction in cellular energetics, and suggesting that mitochondria are the most sensitive organelles to Tl (Rodríguez-Mercado et al., 2019).

## 3 Mutations induced by thallium

Although the effects of Tl on genetic material have yet to be fully explored, it is known that Tl^+^ is molecularly more stable than Thallic cation (Tl_3_^+^), which has a strong oxidizing capacity (Harris and Messori, 2002). Under experimental UV irradiation and the presence of molecular oxygen, Tl^+^ can be oxidized to Tl_3_^+^, which in turn, can oxidize guanine residues on DNA to 8-oxoguanine to induce mutagenic effects. Thus, Tl compounds likely exert changes in cell-cycle progression (Nowicka, et al., 2013; Rodríguez-Mercado et al., 2019).

Zasukhina et al. (1983) demonstrated with hydroxyapatite chromatography assays that increased doses of thallium carbonate added for 24 h to rodent embryonic fibroblast cells induced single-stranded DNA breaks. In addition, the mutagenic potential of Tl was tested using the dominant lethal test, which consists of chronically Tl exposed males crossed with unexposed females. The effects were characterized by an increase in the total number of embryonic deaths. However, these results must be confirmed using a better sample size. Additionally, these authors explored the Vaccinia virus (Lister) strain upon exposure to Tl carbonate, and the induced lethal mutations on DNA virus were estimated by comparing the virus titer between the control and thallium-treated group, showing that thallium carbonate reduced virus survival and suggesting that a significant rate of mutability resulting in non-viable viruses was induced by this genotoxic agent.

Several groups have demonstrated that thallium sulfate administered to chick embryos reduced the growth of the long bones and induced areas of necrotic chondrocytes containing low levels of acid mucopolysaccharide. It is likely that Tl sulfate can induce mutations in an enzyme essential for acid mucopolysaccharide synthesis, or in constitutive membrane proteins of chondrocytes, thus altering the release of the acid mucopolysaccharide. These mutations might be related to the fact that the resulting progenies of the Tl-treated embryos had short-limbed dwarfism even when the parents were healthy (Léonard and Gerber, 1997).

## 4 Epigenetic changes

Exogenous DNA damage occurs when environmental, physical, and chemical agents affect the DNA integrity, as it occurs with exposure to UV and ionizing radiation, alkylating agents, external DNA-binding molecules, and crosslinking agents (Chatterjee and Walker, 2017). Nowicka et al. (2013) reported that Tl can interact with selected oligonucleotide gene sequences such as those present in the human gene OGG1 responsible for repairing DNA damage. This gene encodes for the enzyme responsible for the excision of 8- oxoguanine, a mutagenic base byproduct that is generated by exposure to reactive oxygen species. Using cyclic voltammetry assays, these authors showed that under basal conditions, nitrogen N-7 in guanine structure can be oxidized. The binding of this residue to Tl(I) leads to the loss of this electroactivity. The resulting binding failed to induce changes in DNA conformation, but increased the probability of a concurrent introduction of an incorrect base and propagation in subsequent rounds of DNA replications. Authors also demonstrated that the addition of guanine-enriched telomeric sequences in one of the strands of the DNA helix limited the interaction of Tl with dsDNA. Despite these results, authors concluded that the presence of monovalent thallium cations did not alter the DNA double helix nor induced its oxidization, though the possibility of introducing and preserving mutations on DNA sequence remained.

Although this study concluded that Tl did not alter the DNA conformation substantially, this element should be considered a DNA-damaging agent due to its ability to form numerous bonds within the major groove of DNA, to methylate guanine profile, to elicit 8-oxo-guanine lesions, and to favor single and double-strand breaks, as well as its interstrand guanine crosslinking effect. These abnormalities trigger specific DNA repairing pathways such as repairing replication mismatch errors, base-excision enzyme machinery, repairing stranded breaks in the DNA backbone, and nucleotide excision to eliminate intra-strand crosslinks (Chatterjee and Walker, 2017). The interaction of Tl, especially with the guanine residues on DNA, strongly suggests that Tl might be involved in the modulation of methylation of CpG islands present in promoter regions of several genes subjected to epigenetic control to regulate their transcription as it is known to occur during developmental programming of fetal growth to adult organisms, preventing or allowing the access of transcription factors or protein complexes to DNA. Alternatively, it has been reported that epigenetic control is responsible for silencing tumor suppressor genes associated with carcinogenic processes (John and Rougeulle, 2018; Nishiyama and Nakanishi, 2021), though it is necessary to perform more studies to confirm these hypotheses.

Mitochondrial DNA is susceptible to oxidative damage when protective histones and effective DNA repair mechanisms are absent. Histones modulate the cellular accessibility of nuclear factors to DNA. An excess in histone proteins supply has been invoked in increased DNA damage susceptibility and blocking the DNA repair process by interfering with the homologous recombination-mediated DNA repair mechanisms (Kumar et al., 2020). The mitochondrial DNA copy number is altered during physiological malfunctions or adverse environmental conditions. This parameter is considered a cellular marker that indicates deficits in the energy production balance required to maintain optimal cellular and physiological functions. Wu et al. (2019) analyzed the urine of pregnant women exposed to environmental thallium. These investigators demonstrated a significant decrease in the amounts of mitochondrial DNA copy numbers, mainly during the first trimester. In addition, this group determined the neonatal relative telomere length by quantitative real-time polymerase chain reaction assays in cord blood leukocytes. The relative telomere length is a hallmark of cell aging and it has been involved in preserving genomic stability and chromosomal integrity (Wu et al., 2021). These results suggest a close relationship between prenatal exposure to Tl and decreased mitochondrial DNA copy number and shortened neonatal telomere length due to epigenetic changes in histone proteins, such as H3 and H4. Table 1 summarizes the main effects of Tl on DNA damage and epigenetic modifications.

**TABLE 1:** Effects of Thallium on DNA damage and epigenetic modifications.

Modification		Effects	References
DNA	Oxidation of guanine residues on DNA to 8-oxoguanine	Alters DNA replication	Nowicka et al. (2013); Rodrìguez-Mercado et al. (2019)
	Introducing single-stranded DNA breaks	Induces embryonic death	Zasukhina et al. (1983)
Epigenetic	Mutations on OGG1 gen	Affects DNA repairing mechanisms	Chatterjee and Walker (2017)
	Tl interactions with guanine residues on DNA	Alters the methylation pattern of CpG islands in promoter regions	Chatterjee and Walker (2017)
	Decreases in the copy number of mitochondrial DNA	Deficit in energy production balance	Wu et al. (2019)
	Shortening the telomere length due to epigenetic changes on H3 and H4 histone proteins	Alterations in cell aging evoking death mechanisms	Wu et al. (2019)

## 5 Thallium and cancer

The negative effects exerted by Tl on severThe negative effects exerted by Tl on several cell functions, such as cell survival, the introduction of mutations, disruption of DNA methylation control mechanisms, and inducible DNA damages, have been well documented. These abnormalities might trigger several diseases, including cancer. Previously, Tl was shown to exert missense mutations, insertions, translocations on DNA, changes into the DNA backbone, histone profile modifications, and chromatin architecture, to name a few, which can modulate gene expression while reprograming the development and progression of tumor cells (Casalino and Verde, 2020). These studies highlight the ability of Tl to induce some changes in the DNA methylation patterns, especially the methylation of promoter CpG islands and transcriptional silencing of tumor suppressors genes, thus modifying genomic stability and gene expression of key oncogenic genes (Jones and Baylin, 2007; Casalino and Verde, 2020; Nishiyama and Nakanishi, 2021).

Nowicka et al. (2013) reported that Tl(I) can decrease the activity of doxorubicin (DOX), a popular anticancer drug directed to DNA that targets a group of three guanines. These results suggest that Tl(I) interferes with the metabolism of certain antineoplastic agents, modifying or inhibiting their anti-carcinogenic action and therefore allowing the progression of tumorigenesis (Léonard and Gerber, 1997). In addition, it has been demonstrated that acute *in vivo* administration of Tl induces dysfunctions in the assembly of ribosomes due to structural changes observed in the endoplasmic reticulum of hepatocytes, decreasing the activity of several drug-metabolizing enzymes dependent on cytochrome P-450. Consequently, the Tl-induced effects might confer carcinogenic susceptibility to certain cell populations (Fowler et al., 1993).

Further studies are warranted using these two oxidation states of thallium, as thallium (I) sulfate and thallium (III) chloride showed different effects in diminishing the mitotic index in human peripheral lymphocytes at all tested concentrations (Rodríguez-Mercado et al., 2017). These studies might contribute to a better understanding as to whether the intercalating property of Tl to DNA is more potent in affecting the survival and proliferation rate of neoplastic cells.

On the other hand, Tl has been used for tumor imaging due to its similarity to alkali metals such as cesium (Nadel, 1993; Kumar et al., 2017). However, the exact mechanism of Tl specificity to tumor cells remains unclear. Noteworthy, other studies have demonstrated that Tl might show anti-tumor activity. Hart and Adamson (1971) demonstrated that Tl trichloride (2–5 mg/kg) increased more than 700% the survival of rats implanted with the ascites form of Walker 256 carcinosarcoma compared to the control group.

Recently, 3 Tl(III) synthetic complexes were obtained showing selective cytotoxic effects against the cancer cell lines A375 (derived from human melanoma) and HT29 (derived from human colon adenocarcinoma), though these inhibitory effects were weaker on the normal fibroblast cell line HFF (isolated from human foreskin). The cytotoxic activity of these Tl derivatives was determined by MTT assays reporting a significant reduction of cell viability in these cell lines after treatment with thallium complexes, particularly by C1 and C3 compounds. The cellular uptake of Tl compounds was analyzed revealing that the cellular concentration of Tl was lower in normal cells than in cancer cells. When the generation of reactive oxygen species (ROS) was analyzed in A375 cells, all three complexes increased the ROS levels, which contrasted with much lower levels in HFF cells. ROS generation was accompanied by the reduction of mitochondrial membrane potential in A375 cells, whereas in Tl -treated HFF cells no significant changes in this parameter were observed. By flow cytometry assays on A375 cells, it was shown that Tl complexes induced arrest in the G2-M cell cycle phase with the consequent induction of apoptosis. The cell cycle did not show significant changes in HFF cells. The type of cell death induced in this cell line depicted increases in the late apoptosis pattern compared to Tl-untreated A375 cells. The suggested apoptotic pathway activated in this cell line was confirmed by western blotting assays which showed an increase in p53 protein, Bax upregulation, Bcl-2 down-regu ation, cytochrome c release, and augmented expression of procaspase-9, and 3 (Abdolmaleki et al., 2021).

Our group recently demonstrated that Tl exerted cytotoxic effects in two glioblastoma cell lines (C6 and U373) exposed to increased concentrations of thallium (I) acetate. By means of atomic absorption assays, it was shown to accumulate in the cytoplasm of C6 and U373 cells, supporting an efficient uptake mechanism. The cytotoxic effect of Tl in these cells was spectrophotometrically determined by the incorporation of the neutral red dye, showing that cell viability decreased in a concentration-dependent manner in both cell lines compared to primary cell cultures. The loss of confluence and morphological changes in cancer cells was determined by light microscopy. The type of cell death observed in these neoplastic cells was assayed by the incorporation of ethidium bromide and the enzymatic metabolism of the acridine orange. Epifluorescence assays demonstrated chromatin condensation and nuclear fragmentation, as well as the formation of apoptotic bodies compared to the control group and brain primary cultures. Propidium iodide staining assays revealed that Tl exposure evoked changes in the cell cycle in C6 cells, where a prominent G1 phase was induced, while U373 cells were arrested in the subG1 phase. This study established that Tl was able to exert toxic effects preferentially in tumor cells compared to primary brain cultures (Rangel-López et al., 2022).

Altogether, the above-described evidence establishes a dual scenario where, in some instances, Tl might be responsible for mutagenesis and alterations possibly leading to tumorigenesis, while opposite to these changes, Tl might exert direct toxic effects on tumor cells due to its affinity for these cells. Studies clarifying the effects of this metal on tumor phenotypes are warranted, searching for the conditions by which Tl may act as a tumorigenic or antitumor element.

## 6 Concluding remarks

Although cytotoxic, mutagenic, and genotoxic effects have been ascribed to Tl decades ago, it was not considered a carcinogenic element due to the lack of more relevant human and animal studies (IARC, 1989). Here, we noted several studies which suggest that Tl has damaging properties like those observed by other metals, such as beryllium (Gordon and Bowser, 2003), cadmium (Waisberg et al., 2003), lead (García- Lestón et al., 2010) and mercury (Aschner and Aschner, 1990; Ajsuvakova et al., 2020). It has been documented that the adverse effects of Tl in living organisms are a consequence of chronic exposure, and these elevated concentrations may be a consequence of anthropogenic activities, such as mining, releasing this metal into the environment. Once inside cells, it has been suggested that mitochondria are the main target for Tl toxicity, affecting cell viability by activating ERK and caspase-3 proteins involved in the apoptotic pathway, as it has been reported for mercury (Aschner and Carvalho, 2019). Combined, these harmful features evoke DNA damage that cells try to offset through base excision repair, nucleotide excision repair, mismatch repair, homologous recombination, and non-homologous end joining, which are active throughout different stages of the cell cycle. These repairing processes and DNA tolerance pathways are essential to preserving the genetic stability in cells (Chatterjee and Walker, 2017). This scenario represents a relevant challenge for researchers and mandates additional studies to further characterize the adverse effects of Tl.

In addition, it is important to consider that Tl not only exerts negative effects on human health but its chemical resemblance as a potassium analog and its ability to be taken up and accumulate in cancer cells at higher rates than normal cells have allowed for its use as the radioisotope 201TI in imaging as an accurate radiotracer to detect and delimit tumors (Jo et al., 2020; Poudyal et al., 2022).

Regarding the antineoplastic effects induced by Tl compounds, it seems that the ROS levels generated by the administration of this metal induce more cytotoxic effects in neoplastic cells than in normal cells, suggesting that the cellular levels of ROS play a crucial role in inducing cancer cell death. Finally, it has been demonstrated that Tl has a special affinity for cancer cells likely due to the mimicry between Tl to potassium (Abdolmaleki et al., 2021; Rangel-López et al., 2022). Considering its biochemical and physical properties, its effects on DNA damage, and the energetic requirements of neoplastic cells future studies should focus on strategies to reduce the toxic effects of Tl and to design novel thallium-based drugs to inhibit the development and progression of several cancer types.

**FIGURE 1:**
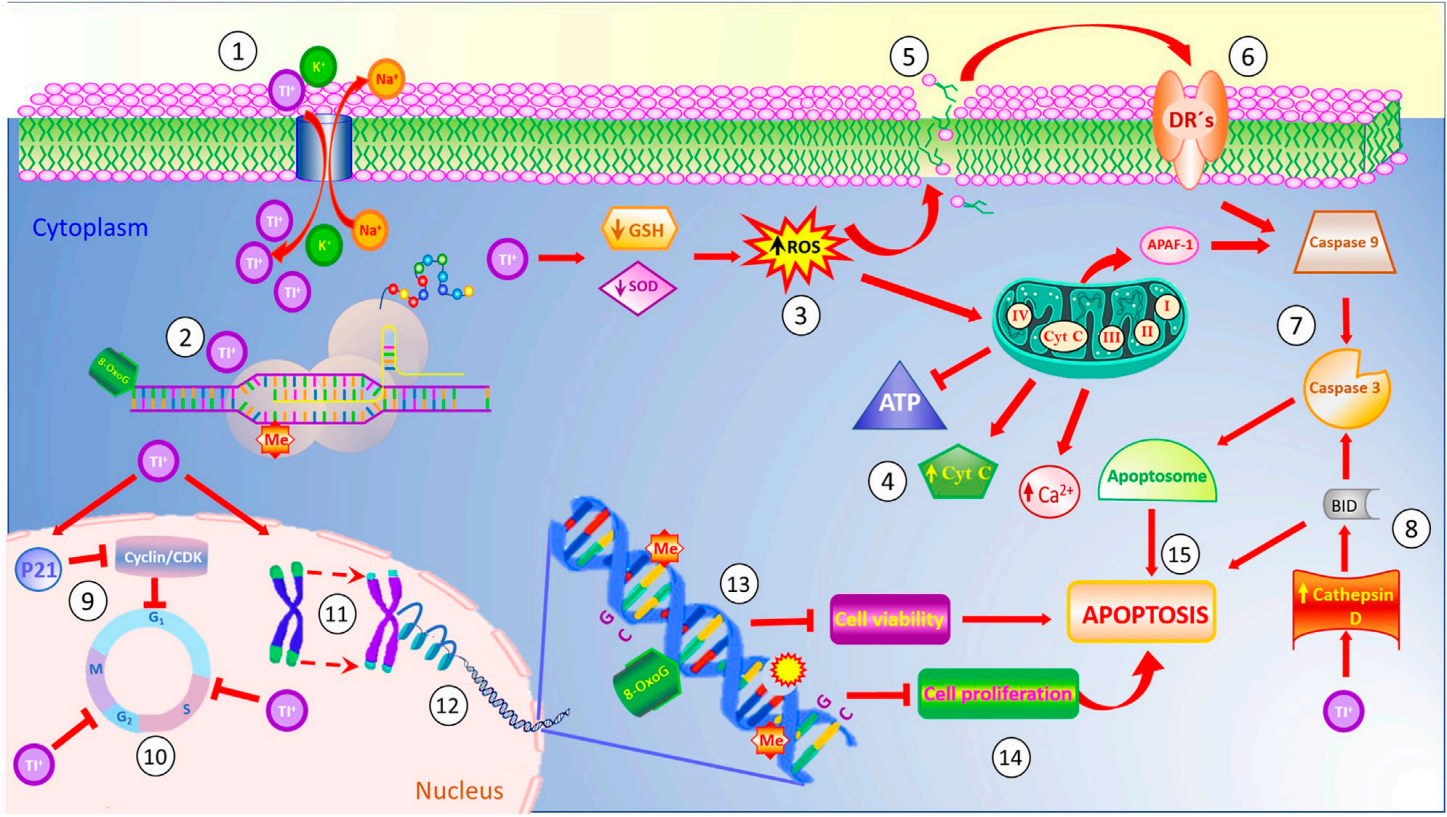
Thallium affects the function of the Na^+^/K^+^ pump ATPase due to its chemical similarity to potassium (1). Once inside the cell, Tl binds to sulfhydryl-enriched proteins affecting their normal functions, inhibiting the stability of ribosomes and protein synthesis (2). Also, cytoplasmic levels of Tl induce poor antioxidant responses, thus decreasing the levels of reduced glutathione (GSH) and superoxide dismutase (SOD), and generating high levels of reactive oxygen species (ROS) (3). These reactive species affect the mitochondrial membrane permeability, causing disruption in the electron transport chain, releasing Ca^+^ and cytochrome C to the cytoplasm, and inhibiting the generation of the energetic molecule of ATP (4). Similarly, high levels of ROS evoke membrane damage via increased lipoperoxidation (5), which, in turn, release factors activating the membrane death receptors (6) and triggering the caspases cascade (7). Cytoplasmic Tl increases the Cathepsin D levels which activate the BID protein involved in the apoptotic pathway (8). On the other hand, Tl also disturbs cell cycle progression (9,10), decreases telomere length (11), and affects the integrity of nucleosomes (12). Inside the DNA molecule, thallium induces the generation of the mutagenic 8-oxo guanine, disturbs the expression of CpG islands in promoter gene sequences, induces DNA breaks, and modifies the methylation pattern of the DNA (13). Combined, these effects decrease cell viability and cell proliferation (14), evoking cell death by apoptosis (15).

Finally, Figure 1 summarizes the most relevant effects induced by cellular uptake of Tl. Since the Tl ion resembles potassium due its ionic radii, the sodium-potassium pump takes thallium instead of potassium (Álvarez-Barrera et al., 2019). Once inside the cell, Tl binds to cellular proteins, enzymes and organelles involved in several physiological functions. The function of ribosomes is altered by binding of Tl to enriched-sulfhydryl groups in their structure, affecting the protein synthesis (Chou and Lo, 2019), and the resulting proteins show misfolding mistakes and/or mutated amino acid sequences. Similarly, Tl decreases glutathione synthesis and reduces the levels of superoxide dismutase (SOD), thus interfering with cellular antioxidant mechanisms and inducing the generation of reactive oxygen species (ROS) (Rodríguez-Mercado and Altamirano-Lozano, 2013). The increase in ROS levels elicits membrane damage by lipoperoxidation, which in turn, generates death signals that are transduced by membrane Death Receptors (DR’s). These receptors trigger the apoptotic pathway mediated by caspases 9 and 3 through the apoptosome complex. Similarly, Tl induces an increase in Cathepsine D levels which activates the protein BID, involved in the intrinsic pathway of apoptosis (Hanzel et al., 2012). In addition, secondary to increased ROS levels induced by intracellular Tl, damage to mitochondria ensues, including electron transport chain disruption. In turn, ATP levels decrease and Ca^+^ cytoplasmic levels increase, leading to the release of cytochrome c and activation of apoptotic protease activating factor-1 (Apaf- 1) (Fani et al., 2016; Abdolmaleki et al., 2021). At the nuclear level, Tl alters the expression of p21 which inhibits the cyclin/CDK complex, blocking the G1/S and G2/M transitions, thus disturbing the cell cycle (Chia et al., 2005), In addition, Tl induces telomere shortening and modifies epigenetic patterns due the structural modifications of histones H3 and H4 from nucleosomes and the sites susceptible of methylations (ME), thus eliciting chromosomal aberrations (Wu et al., 2021). At the DNA level, Tl elicits DNA breaks and favors dysfunctions on enzymatic DNA repairing mechanisms due to the conversion of guanine residues to the mutagenic 8-oxo guanine, affecting the expression of CpG enriched sequences (Nowicka et al., 2013). Combined, these malfunctions inhibit cell viability and cellular proliferation eliciting the apoptosis of the affected cells (Rodríguez-Mercado and Altamirano-Lozano, 2013).
